# Comprehensive analysis of single-repeat R3 MYB proteins in epidermal cell patterning and their transcriptional regulation in Arabidopsis

**DOI:** 10.1186/1471-2229-8-81

**Published:** 2008-07-21

**Authors:** Shucai Wang, Leah Hubbard, Ying Chang, Jianjun Guo, John Schiefelbein, Jin-Gui Chen

**Affiliations:** 1 Department of Botany, University of British Columbia, Vancouver, BC V6T 1Z4, Canada; 2 Department of Molecular, Cell, and Developmental Biology, University of Michigan, Ann Arbor, MI 48109, USA

## Abstract

**Background:**

Single-repeat R3 MYB transcription factors are critical components of the lateral inhibition machinery that mediates epidermal cell patterning in plants. Sequence analysis of the Arabidopsis genome using the BLAST program reveals that there are a total of six genes, including *TRIPTYCHON *(*TRY*), *CAPRICE *(*CPC*), *TRICHOMELESS1 *(*TCL1*), and *ENHANCER of TRY and CPC 1*, *2*, and *3 *(*ETC1*, *ETC2 *and *ETC3*) encoding single-repeat R3 MYB transcription factors that are approximately 50% identical to one another at the amino acid level. Previous studies indicate that these single-repeat R3 MYBs regulate epidermal cell patterning. However, each of the previous studies of these single-repeat R3 MYBs has been limited to an analysis of only a subset of these six genes, and furthermore, they have limited their attention to epidermal development in only one or two of the organs. In addition, the transcriptional regulation of these single-repeat R3 MYB genes remains largely unknown.

**Results:**

By analyzing multiple mutant lines, we report here that TCL1 functions redundantly with other single-repeat R3 MYB transcription factors to control both leaf trichome and root hair formation. On the other hand, ETC1 and ETC3 participate in controlling trichome formation on inflorescence stems and pedicles. Further, we discovered that single-repeat R3 MYBs suppress trichome formation on cotyledons and siliques, organs that normally do not bear any trichomes. By using Arabidopsis protoplast transfection assays, we found that all single-repeat R3 MYBs examined interact with GL3, and that GL1 or WER and GL3 or EGL3 are required and sufficient to activate the transcription of *TRY*, *CPC*, *ETC1 *and *ETC3*, but not *TCL1 *and *ETC2*. Furthermore, only *ETC1*'s transcription was greatly reduced in the *gl3 egl3 *double mutants.

**Conclusion:**

Our comprehensive analysis enables us to draw broader conclusions about the role of single-repeat R3 MYB gene family than were possible in the earlier studies, and reveals the genetic basis of organ-specific control of trichome formation. Our findings imply the presence of multiple mechanisms regulating the transcription of single-repeat R3 MYB genes, and provide new insight into the lateral inhibition mechanism that mediates epidermal cell patterning.

## Background

There are a total of six genes in the Arabidopsis genome that encode a unique subfamily of MYB transcription factors, namely single-repeat R3 MYB transcription factors. These transcription factors, including *TRIPTYCHON *(*TRY*) [1,2], *CAPRICE *(*CPC*) [3], *TRICHOMELESS1 *(*TCL1*) [4], *ENHANCER of TRY and CPC 1*, *2*, and *3 *(*ETC1*, *ETC2 *and *ETC3 (CPL3)*) [5-9], are characterized by their short sequence (75–112 amino acids) and consist largely of the single MYB domain (e.g. without other predicted motifs). It is generally believed that these single-repeat R3 MYB transcription factors mediate lateral inhibition during epidermal patterning. In general, these single-repeat R3 MYB transcription factors act as negative regulators for trichome formation in shoots, but as positive regulators for root hair formation in roots.

T-DNA insertion mutants are available for each of these six single-repeat R3 MYB genes. Among them, only single loss-of-function mutants for *TRY*, *CPC *and *TCL1 *display major defects in trichome and/or root hair cell specification [1-4], whereas loss-of-function alleles of *ETC1*, *ETC2 *or *ETC3 *cause little or no phenotypic effect [5-9]. The analysis of double and triple mutants indicated that *ETC1 *and *ETC3 *can function redundantly with *TRY *and *CPC *to control leaf trichome and root hair formation [5,6,9], and that *ETC2 *functions redundantly with *TRY *and *CPC *to control trichome formation on petioles [7]. Further, *CPC *functions redundantly with *TCL1 *to control trichome formation on the inflorescence stems and pedicels [4]. However, a role of TCL1 in leaf trichome and root hair formation has not been established.

Available evidence suggests that single-repeat R3 MYB transcription factors, a WD40-repeat protein, TRANSPARENT TESTA GLABRA1 (TTG1) [10,11], an R2R3 MYB-type transcription factor, GLABRA1 (GL1) [12] or WEREWOLF (WER) [13-15], a bHLH transcription factor, GLABRA3 (GL3) or ENHANCER OF GLABRA3 (EGL3) [16,17], and a homeodomain protein, GLABRA2 (GL2) [18,19], regulate trichome and/or root hair formation (reviewed in [20-22]). Based on the results of yeast-two-hybrid interaction assays, it has been proposed that TTG1, GL1 or WER, and GL3 or EGL3 form an activator complex to induce *GL2 *expression [16]. The single-repeat R3 MYB transcription factors are proposed to move from a trichome precursor cell to its neighboring cell (in the shoot epidermis) or from an N cell to an H cell (in the root epidermis) to compete with GL1 or WER for binding GL3 or EGL3, thus limiting the activity of the activator complex [2,3,20-24]. Recently, we showed that one of the single-repeat R3 MYB transcription factors, TCL1, can directly suppress the transcription of *GL1 *[4], providing an additional loop of regulation of the activity of the proposed activator complex by single-repeat R3 MYB transcription factors.

It has been proposed that the same activator complex that activates *GL2 *can also activate the expression of single-repeat R3 MYB genes (reviewed in [20-22]), but so far only *CPC *has been identified as a direct target gene for WER [25,26], and GL3 has been shown to be recruited to the promoter region of *CPC *and *ETC1 *[27].

To gain new insight into the role of single-repeat R3 MYB transcription factors in controlling epidermal development, we conduct a comprehensive analysis of the single-repeat R3 MYB gene family. By generating and analyzing higher order mutants among six single-repeat R3 MYB genes, we identified previously unrecognized roles of single-repeat R3 MYB transcription factors in the regulation of trichome and root hair formation. We demonstrate that TCL1 participates in the control of leaf trichome and root hair formation, and that ETC1 and ETC3 have a role in regulating trichome formation on the inflorescence stems and pedicles. We also discover that single-repeat R3 MYBs normally suppress trichome formation on siliques and cotyledons. By using an Arabidopsis protoplast transfection system, we show that cotransfection of GL1 or WER, with GL3 or EGL3, is sufficient to activate the transcription of *TRY*, *CPC*, *ETC1 *and *ETC3*, but not *ETC2 *and *TCL1*. Our results suggest that although the six single-repeat R3 MYB genes have largely overlapping functions in controlling epidermal development, the transcriptional regulation of these single-repeat R3 MYB genes involves distinct mechanisms.

## Results

### Single-repeat R3 MYB transcription factors in Arabidopsis

**Figure 1:**
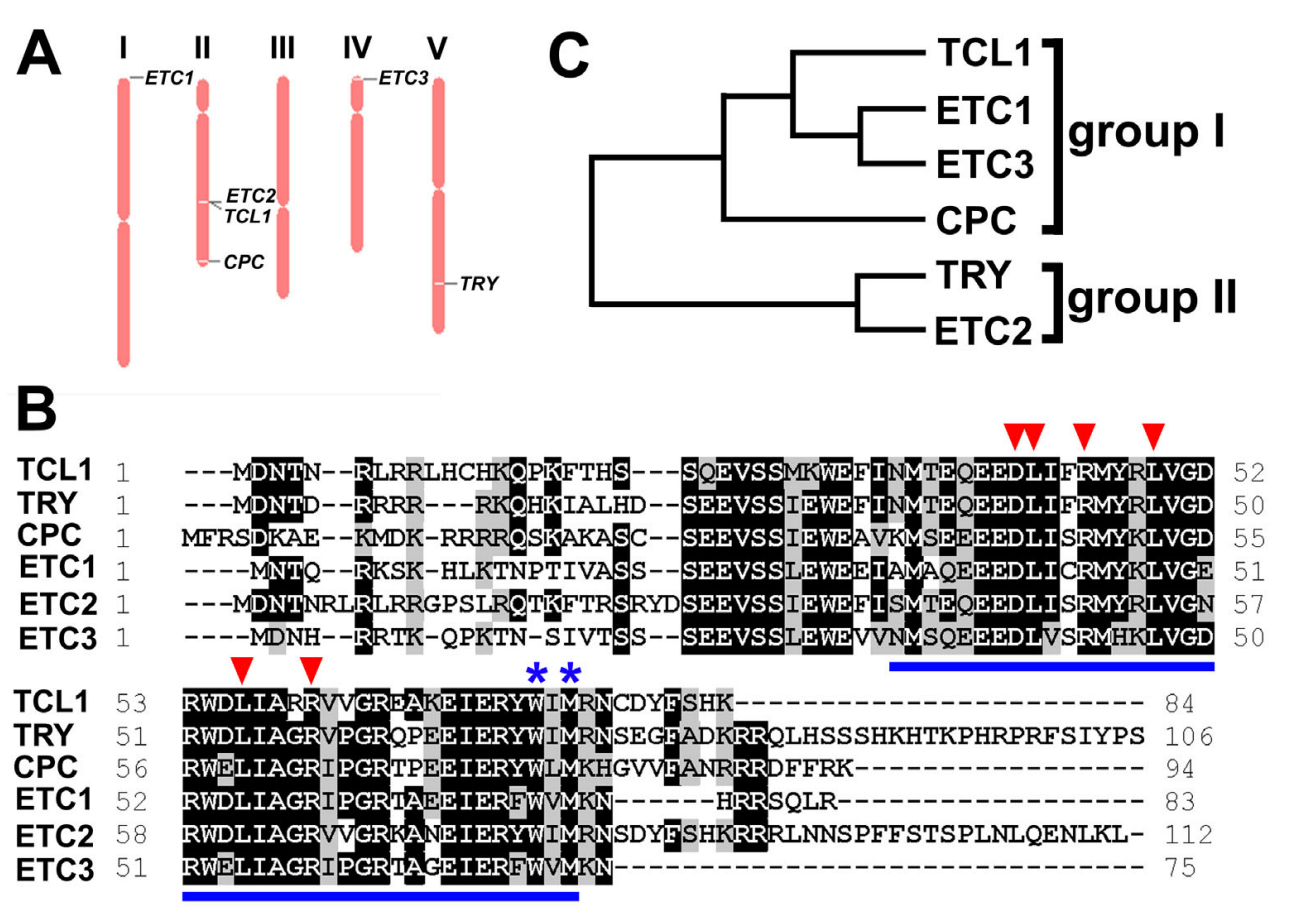
**Single-repeat R3 MYB transcription factors in Arabidopsis**. (**A**) Chromosome location of six single-repeat R3 MYB genes. (**B**) Amino acid sequence alignment of single-repeat R3 MYB transcription factors. Identical amino acids are shaded in black and similar amino acids in gray. The single R3 MYB domain is indicated with think lines underneath. The amino acid signature [D/E]L × 2 [R/K] × 3L × 6L × 3R [28] that is required for interacting with R/B-like BHLH transcription factors is indicated by arrowheads on the top of amino acids. The amino acids within the MYB domain that are crucial for cell-to-cell movement of CPC [29] are indicated by asterisks on the top of amino acids. (**C**) Phylogenetic analysis of the single-repeat R3 MYB transcription factors. The phylogenetic tree using the entire amino acid sequence was generated using software AliBee – Multiple alignment Release 2.0

Sequence analysis of the Arabidopsis genome using the BLAST program  with the entire amino acid sequence of TCL1 reveals that there are a total of six genes, including *TRY*, *CPC*, *TCL1*, *ETC1*, *ETC2 *and *ETC3*, encoding single-repeat R3 MYB transcription factors that are approximately 50% identical to one another at the amino acid level. These six genes are unevenly distributed in four of the five chromosomes of Arabidopsis (Figure 1A). Among them, *ETC2 *(At2g30420) and *TCL1 *(At2g30432) are tandem genes located in chromosome II (Figure 1A). The amino acid signature [D/E]L × 2 [R/K] × 3L × 6L × 3R [28] that has been shown to be required for interacting with R/B-like bHLH transcription factors is completely conserved in all single-repeat R3 MYB transcription factors (Figure 1B). The amino acids within the MYB domain that have been shown to be crucial for the cell-to-cell movement of CPC [29] are also entirely conserved in all single-repeat R3 MYBs (Figure 1B). Phylogenetic analysis using the entire amino acid sequence suggested that these six single-repeat R3 MYB transcription factors can be divided into two groups, with TCL1, ETC1, ECT3 and CPC in group I, and TRY and ETC2 in group II (Figure 1C).

### *TCL1 *affects both leaf trichome and root hair formation

**Figure 2:**
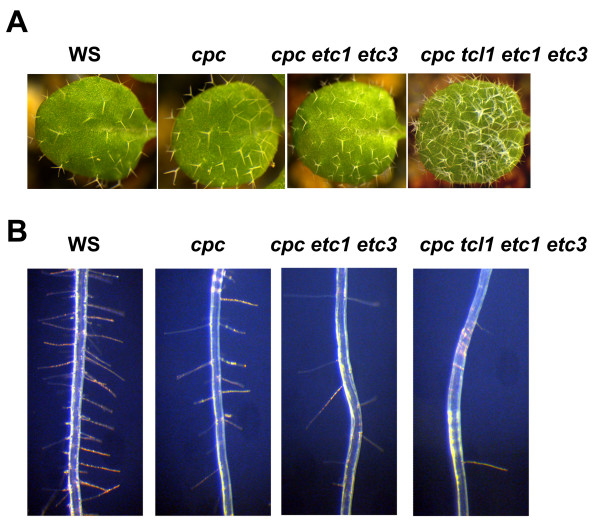
**TCL1 participates in the control of both leaf trichome and root hair formation**. (**A**) TCL1 functions redundantly with other single-repeat R3 MYB transcription factors to control trichome formation on leaves. Pictures were taken from 2-week-old, soil-grown seedlings. (**B**) TCL1 functions redundantly with other single-repeat R3 MYB transcription factors to control root hair formation. Pictures were taken from 7-day-old seedlings grown on vertically orientated 1/2 MS plates.

Among the six single-repeat R3 MYB transcription factors, TRY and CPC have been shown to be involved in both leaf trichome and root hair formation [1-3,30], while ETC1 can function redundantly with TRY and CPC to control leaf trichome and root hair formation [5,6] and ETC2 functions redundantly with TRY and CPC to control trichome formation on petioles [7]. Recently, it has been shown that ETC3 functions redundantly with single-repeat R3 MYBs to regulate both trichome and root hair formation [9]. However, a role of TCL1 in leaf trichome and root hair formation has not been established, although previously, we showed that TCL1 controls trichome formation on the inflorescence stems and pedicles [4]. To test if TCL1 participates in the control of leaf trichome and root hair formation, we generated double, triple and quadruple mutants among all single-repeat R3 MYB genes in group I including *TCL1*, *ETC1*, *ETC3 *and *CPC *(Figure 1C). Consistent with previous report [2], the *cpc *single mutant has significantly increased trichome number on leaves (Figure 2A, Table 1). All other single mutants of group I genes examined are indistinguishable from wild-type plants (Table 1). We found that although double and triple mutants including *cpc *(e.g. *cpc etc1 etc3*) are similar to the *cpc *single mutant, and double and triple mutants between *etc1, etc3*, and *tcl1 *are similar to wild type (Table 1), the *cpc etc1 etc3 tcl1 *quadruple mutant has dramatically increased trichome number on leaves than *cpc *single mutant and *cpc etc1 etc3 *triple mutant (Figure 2A, Table 1), indicating that TCL1 is involved in the regulation of leaf trichome formation.

**Table 1:** Leaf trichome production in wild-type, mutants, and transgenic Lines. Values indicate mean ± standard deviation of at least ten rosette leaves for each line.

Genotype	Number of Trichomes Per Leaf	Frequency of Trichome Clusters (%)
WT (Col)	33.5 ± 6.0	0
WT (WS)	31.9 ± 5.7	0
*cpc-1*	56.5 ± 10.2 *	0
*try-29760*	38.4 ± 7.2	12.1 *
*tcl1-1*	26.8 ± 5.1	0
*etc1-1*	40.8 ± 7.2	0
*etc2-2*	32.7 ± 4.2	0
*etc3-1*	31.3 ± 4.0	0
*etc3-1 cpc-1*	59.5 ± 6.6 *	1.2 *
*etc3-1 try-29760*	31.3 ± 4.2	8.8 *
*etc3-1 tcl-1*	33.9 ± 5.0	0.3
*etc3-1 etc1-1*	28.8 ± 4.2	0.5
*etc3-1 etc2-2*	35.5 ± 6.1	0
*tcl1-1 cpc-1*	49.9 ± 8.5 *	2.4 *
*tcl1-1 try-29760*	32.5 ± 6.3	9.5 *
*cpc-1 try-29760*	156 ± 28 *	89 *
*cpc-1 try-29760 tcl1-1*	135 ± 36 *	93 *
*cpc-1 try-29760 etc1-1*	190 ± 42 *	99 *
*cpc-1 tcl1-1 etc3-1*	46.6 ± 7.8 *	1.4 *
*cpc-1 etc1-1 etc3-1*	51.9 ± 8.7 *	7.0 *
*cpc-1 tcl1-1 etc1-1*	40.5 ± 3.2	0.4
*etc3-1 try-29760 tcl1-1*	31.6 ± 4.5	11.5 *
*cpc-1 tcl1-1 etc1-1 etc3-1*	106 ± 23 *#	16 *
*cpc-1 try-29760 tcl1-1 etc1-1*	182 ± 38 *	98 *
*35S:HA-ETC3*	0 ± 0 *	0
*P*_*ETC3*_*:ETC3-GFP*	0 ± 0 *	0

* p < 0.05, relative to the corresponding wild type line.

# p < 0.05, relative to the *cpc-1 etc1-1 etc3-1 *line.

Subsequently, we examined root hair formation in these mutants. As shown in Figure 2B and Table 2, *cpc *single mutant had reduced root hair formation, consistent with previous reports [3,30]. Compared with the *cpc *single mutant, the *cpc etc1 etc3 *triple mutant produced fewer root hairs (Figure 2B, Table 2). Moreover, *cpc etc1 etc3 tcl1 *quadruple mutant produced significantly fewer root hairs than *cpc etc1 etc3 *triple mutant (Figure 2B, Table 2), indicating that TCL1 also participates in the regulation of root hair formation.

**Table 2:** Root-hair and non-hair cell specification in the root epidermis of wild-type, mutant, and transgenic Lines.

Genotype	Hair cells in epidermis (%)	H cell position		N cell position	
			
		Hair cells (%)	Non-hair cells (%)	Hair cells (%)	Non-hair cells (%)
WT (Col)	41.6 ± 2.9	97.1 ± 1.7	2.9 ± 1.7	0.5 ± 1.1	99.5 ± 1.1
WT (WS)	40.2 ± 4.2	96.2 ± 2.4	3.8 ± 2.4	0.3 ± 0.3	99.7 ± 0.3
*cpc-1*	14.2 ± 2.4 *	24.2 ± 2.7	75.8 ± 2.7	0.5 ± 1.0	99.5 ± 1.0
*try-29760*	40.0 ± 4.9	94.5 ± 3.5	5.5 ± 3.5	1.3 ± 2.0	98.7 ± 2.0
*tcl1-1*	42.7 ± 4.4	95.8 ± 2.9	4.2 ± 2.9	0.2 ± 0.6	99.8 ± 0.6
*etc1-1*	39.4 ± 3.2	93.8 ± 4.2	6.2 ± 4.2	1.8 ± 1.9	98.2 ± 1.9
*etc2-2*	38.5 ± 5.4	95.9 ± 4.9	4.1 ± 4.9	0 ± 0	100 ± 0
*etc3-1*	39.0 ± 4.1	96.7 ± 3.5	3.3 ± 3.5	1.1 ± 0.6	98.9 ± 0.6
*etc3-1 cpc-1*	16.6 ± 3.3*	27.8 ± 4.2	72.2 ± 4.2	0.5 ± 0.5	99.5 ± 0.5
*etc3-1 try-29760*	39.1 ± 4.9	95.0 ± 3.8	5.0 ± 3.8	1.9 ± 2.1	98.1 ± 2.1
*etc3-1 tcl-1*	41.8 ± 3.0	96.7 ± 2.9	3.3 ± 2.9	1.1 ± 1.0	98.9 ± 1.0
*etc3-1 etc1-1*	40.1 ± 4.3	94.1 ± 5.3	5.9 ± 5.3	1.8 ± 2.5	98.2 ± 2.5
*etc3-1 etc2-2*	38.5 ± 5.5	96.0 ± 3.9	4.0 ± 3.9	0.6 ± 1.0	99.4 ± 1.0
*tcl1-1 cpc-1*	14.8 ± 3.0 *	24.4 ± 4.1	75.6 ± 4.1	0 ± 0	100 ± 0
*tcl1-1 try-29760*	40.5 ± 2.9	93.9 ± 4.0	6.1 ± 4.0	1.2 ± 1.2	98.8 ± 1.2
*cpc-1 try-29760*	0 ± 0 *	0 ± 0	100 ± 0	0 ± 0	100 ± 0
*cpc-1 try-29760 tcl1-1*	0 ± 0 *	0 ± 0	100 ± 0	0 ± 0	100 ± 0
*cpc-1 try-29760 etc1-1*	0 ± 0 *	0 ± 0	100 ± 0	0 ± 0	100 ± 0
*cpc-1 tcl1-1 etc3-1*	20.9 ± 3.9 *	41.7 ± 5.8	58.3 ± 5.8	0.6 ± 1.3	99.4 ± 1.3
*cpc-1 etc1-1 etc3-1*	8.5 ± 2.7 *	16.5 ± 4.0	83.5 ± 4.0	0 ± 0	100 ± 0
*cpc-1 tcl1-1 etc1-1*	22.6 ± 4.1 *	43.2 ± 5.1	56.8 ± 5.1	0.2 ± 0.4	99.8 ± 0.4
*etc3-1 try-29760 tcl1-1*	41.3 ± 4.7	95.1 ± 4.4	4.9 ± 4.4	0.9 ± 1.2	99.1 ± 1.2
*cpc-1 tcl1-1 etc1-1 etc3-1*	3.4 ± 1.9 *#	9.2 ± 2.6	90.8 ± 2.6	0 ± 0	100 ± 0
*cpc-1 try-29760 tcl1-1 etc1-1*	0 ± 0 *	0 ± 3.3	100 ± 0	0 ± 0	100 ± 0
*35S:HA-ETC3*	48.8 ± 5.2	97.7 ± 3.1	2.3 ± 3.1	15.1 ± 3.9	84.9 ± 3.9
*P*_*ETC3*_*:ETC3-GFP*	41.9 ± 4.4	96.1 ± 3.9	3.9 ± 3.9	2.6 ± 2.2	97.4 ± 2.2

Values indicate mean ± standard deviation of at least 10 roots for each line. In all strains, approximately 40% of epidermal cells are in the H position.

* p < 0.05, relative to the corresponding wild type line.

# p < 0.05, relative to the *cpc-1 etc1-1 etc3-1 *line.

### *ETC1 *and *ETC3 *participate in the control of trichome formation on the inflorescence stems and pedicles

Previously, we reported that TCL1 is a major regulator of trichome formation on the inflorescence stem and pedicels because *tcl1 *single mutants displayed ectopic trichome formation on pedicels, and trichome formation on inflorescence stems was no longer restricted to the internodes before the first flower [4]. No other single mutant of the single-repeat R3 MYB gene family displayed ectopic trichome formation on the inflorescence stems or pedicels. We have previously shown that CPC acts synergistically with TCL1 to control trichome formation on these organs [4]. In this study, we wanted to further investigate if any other single-repeat R3 MYBs can also regulate trichome formation on the inflorescence stems and pedicels.

**Figure 3:**
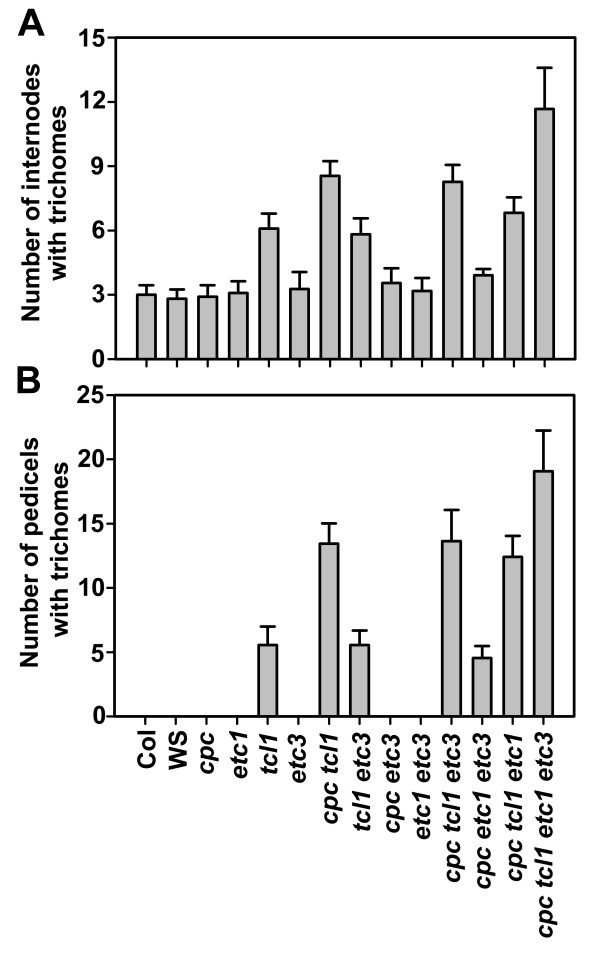
**ETC1 and ETC3 participate in the control of trichome formation on the inflorescence stems and pedicles**. (**A**) Single-repeat R3 MYB transcription factors function redundantly to control trichome formation on the inflorescence stems. (**B**) Single-repeat R3 MYB transcription factors function redundantly to control trichome formation on pedicles. Data represent the mean ± SD of at least 10 plants.

**Figure 4:**
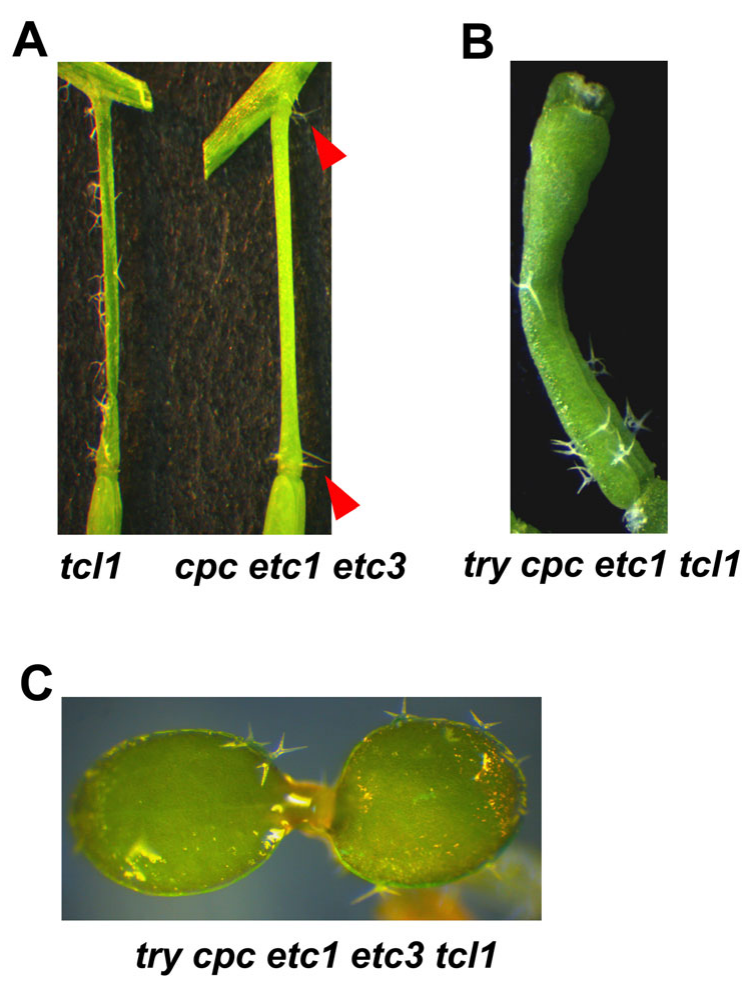
**Single-repeat R3 MYB transcription factors function redundantly to control trichome formation on pedicels, siliques and cotyledons**. (**A**) Comparison of pedicle trichomes in the *tcl1 *single mutant (left) and the *cpc etc1 etc3 *triple mutant (right). Shown are pedicles of the first flowers on the main inflorescence stems. Arrow heads indicate trichomes on both ends of pedicels in *cpc etc1 etc3 *triple mutant. (**B**) Ectopic trichome formation on siliques of the *try cpc etc1 tcl1 *quadruple mutant. No trichomes were formed in the siliques of wild-type plants. (**C**) Ectopic trichome formation on cotyledons of the *try cpc etc1 etc3 tcl1 *quintuple mutant. No trichomes were formed in the cotyledons of wild-type seedlings. Picture was taken from a 5-day-old, light-grown seedling.

We examined trichome formation on the inflorescence stems and pedicles in the double, triple and quadruple mutants of all single-repeat R3 MYB genes in group I including *TCL1*, *ETC1*, *ETC3 *and *CPC*. Compared with the *tcl1 *single mutant or the *cpc tcl1 *double mutant, *cpc etc1 etc3 tcl1 *quadruple mutants have more internodes bearing trichomes (Figure 3A) and more pedicels forming ectopic trichomes (Figure 3B). In addition, ectopic trichome formation was also found on the pedicels of the *cpc etc1 etc3 *triple mutants (Figure 3B, Figure 4A). Unlike the *tcl1 *mutant, whose trichomes are evenly distributed along pedicels, trichomes tend to form at both ends of the pedicels of the *cpc etc1 etc3 *mutant (Figure 4A). These results supported the notion that TCL1 is the major regulator of the single-repeat R3 MYB family controlling trichome formation on the inflorescence stems and pedicels, and that CPC, ETC1 and ETC3 participate in the control of trichome formation on those organs.

### Single-repeat R3 MYBs function redundantly to control trichome formation on siliques and cotyledons

Having generated quadruple mutants with combination of loss-of-function mutations in all group I members of single-repeat R3 MYB genes, including *CPC*, *ETC1*, *TCL1 *and *ETC3*, we sought to generate higher order mutants. The group II small MYBs contains two members, TRY and ETC2 (Figure 1C). As discussed above, *ETC2 *(At2g30420) and *TCL1 *(At2g30432) are tandem genes located in the chromosome II (Figure 1A), which inhibits the generation of sextuple mutants that contain loss-of-function mutations in all six single-repeat R3 MYB genes through classical crosses. Therefore, in this study, the highest order mutant we generated was a quintuple mutant contains loss-of-function mutations in all four members of group I and *TRY *of group II of the single-repeat R3 MYB gene family.

By analyzing the *try cpc etc1 etc3 tcl1 *quintuple mutant and other mutants, we made two discoveries. First, in *try cpc etc1 tcl1 *quadruple mutants, ectopic trichomes were formed on the siliques (Figure 4B). Second, ectopic trichomes were formed on the cotyledons of *try cpc etc1 etc3 tcl1 *quintuple mutant (Figure 4C). Because these organs in wild-type plants do not bear any trichomes, these results indicated that single-repeat R3 MYB transcription factors normally suppress trichome formation on these organs and acts in a highly redundant manner.

### Single-repeat R3 MYBs function redundantly to control trichome cluster formation on leaves and inflorescence stems

**Figure 5:**
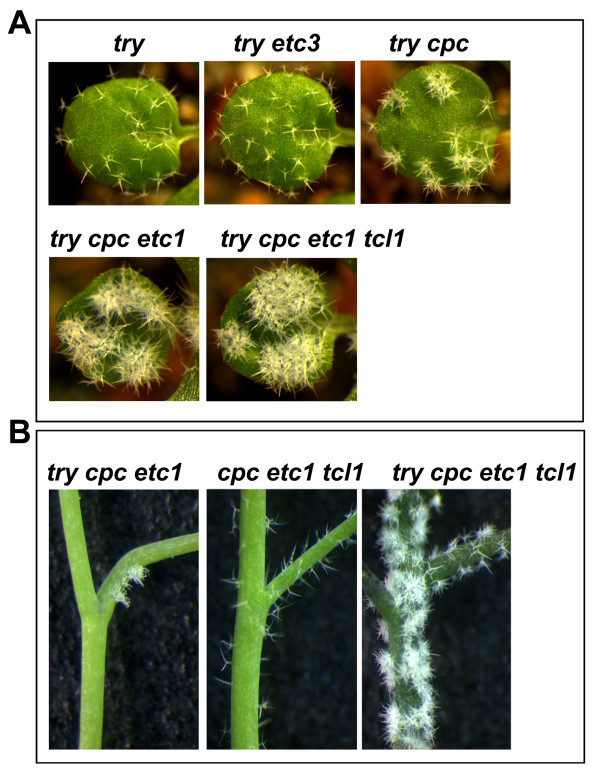
**Single-repeat R3 MYB transcription factors function redundantly to control trichome spacing**. (**A**) Single-repeat R3 MYB transcription factors function redundantly to control trichome cluster formation on leaves. Pictures were taken from 2-week-old, soil-grown seedlings. (**B**) Single-repeat R3 MYB transcription factors function redundantly to control trichome cluster formation on inflorescence stems.

Among all single mutants of the single-repeat R3 MYB genes, only the *try *mutant displays significant trichome clusters on leaves and shoots [1,2]. We wanted to examine whether other single-repeat R3 MYB transcription factors function redundantly with TRY to control trichome spacing. Consistent with that reported previously [6], the size of the trichome clusters on leaves increased significantly from the *try cpc *double mutant to the *try cpc etc1 *triple mutant (Figure 5A). We found that the size of trichome clusters on leaves was further increased in the *try cpc etcl tcl1 *quadruple mutant (Figure 5A), suggesting that TCL1 also contributes to the control of trichome spacing on leaves.

We also found that *etc3-1 *mutation can increase trichome cluster frequency in *cpc *mutant background. As shown in Table 1, the *cpc, etc1, tcl1*, and *etc3 *single mutants are similar to wild type in term of trichome cluster formation. However, in the *cpc etc3 *double mutant, about 1.2% of its trichomes are in clusters, and the number of trichome clusters was increased to about 7% in *cpc etc1 etc3*, and about 16% in *cpc etc1 etc3 tcl1 *(Table 1). These results supported the notion that TRY is the major regulator for controlling trichome cluster formation, and suggested that other single-repeat R3 MYBs contribute to trichome cluster formation by functioning in a redundant manner.

Subsequently, we examined trichome cluster formation on the inflorescence stems. As discussed earlier, TCL1 is a major regulator of trichome formation on the inflorescence stems whereas TRY controls the formation of trichome cluster. As expected, extensive trichome clusters were found on the inflorescence stems of *try cpc etc1 tcl1 *quadruple mutants (Figure 5B).

### Single-repeat R3 MYB transcription factors interact with GL3 in plant cells

Our genetic analyses indicated that single-repeat R3 MYBs regulate trichome and root hair formation in a highly redundant manner. We next wished to study their mechanism of action in trichome and root hair formation. It has been shown that some of these single-repeat R3 MYB transcription factors, including TRY, CPC, ETC1, ETC2 and ETC3, interact with GL3 in yeast cells [7,9,17,24,28]. This property has been proposed to enable the single-repeat R3 MYB transcription factors to compete with GL1 for binding GL3, thus limiting the activity of TTG1-GL3/EGL3-GL1/WER activator complex and inhibiting trichome formation and promoting root hair formation [20-23]. Although TCL1 is likely to behave similarly as TRY, CPC, ETC1, ETC2 and ETC3, a direct test of the interactions between TCL1 and GL3 has not been performed. Further, the interactions in yeast cells (e.g. interaction between GL3 and TRY, CPC, ETC1, ETC2 or ETC3) have not been confirmed in plant cells. Therefore, in this study, we tested the interactions between GL3 and each of these six single-repeat R3 MYB transcription factors using a plant two-hybrid protein-protein interaction system [31].

**Figure 6:**
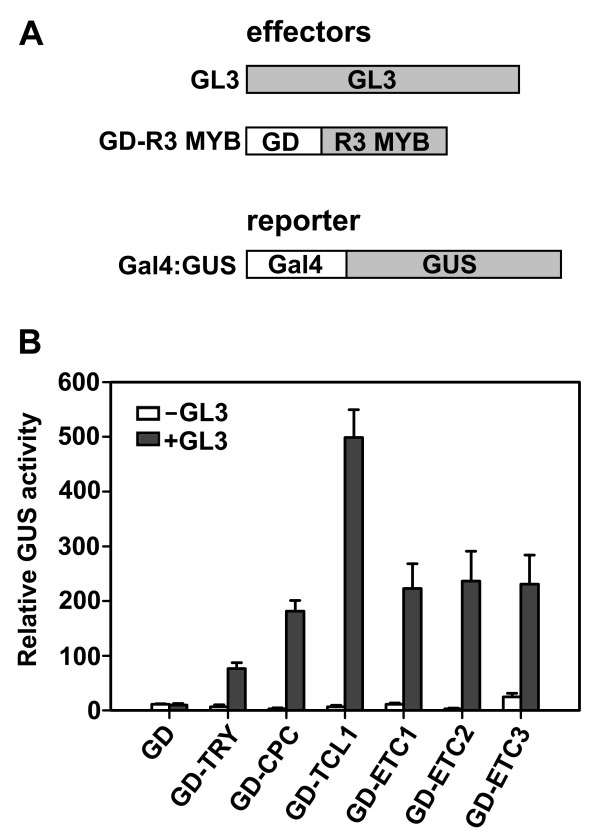
**Single-repeat R3 MYB transcription factors interact with GL3 in plant cells**. (**A**) Effector and reporter constructs used in transfection assays. Effector gene, Gal4 DNA binding domain (GD) fused single-repeat R3 MYB and GL3, and reporter gene, Gal4-GUS, were co-transfected into protoplasts derived from Arabidopsis rosette leaves. (**B**) Relative GUS activity. GUS activity was measured after the transfected protoplasts had been incubated for 20–22 h in the darkness. Data represent the mean ± SD of three replicates.

A Gal4-GUS reporter, together with the effectors GL3 and a Gal4 DNA binding domain (GD) fused single-repeat R3 MYB transcription factor (Figure 6A), were co-transfected into Arabidopsis protoplasts. Although single-repeat R3 MYB transcription factors can be recruited to the promoter region of the reporter gene by GD, they alone cannot activate the expression of the reporter gene due to the lack of a transcription-activating domain. Activation of reporter expression can occur if the small MYB protein interacts with GL3, because GL3 can function as a transcriptional activator (data not shown). Indeed, TRY, CPC, ETC1, ETC2 and ETC3 interacted with GL3 in plant cells (Figure 6B), consistent with the results reported in yeast cells [7,9,17,24,28]. Using this system, we also showed that TCL1 is able to interact with GL3 (Figure 6B). Thus, all six single-repeat R3 MYB transcription factors can interact with GL3 in plant cells, implying that GL3 binding may represent a general mechanism of single-repeat R3 MYB action in inhibiting the activity of the TTG1-GL3/EGL3-GL1 activator complex.

### Transcriptional regulation of single-repeat R3 MYB genes

It has been proposed that the TTG1-GL3/EGL3-GL1 activator complex in shoots not only promotes the transcription of *GL2*, but also promotes the transcription of single-repeat R3 MYB genes, and a similar mechanism has been proposed to operate in roots with WER replacing GL1 (reviewed in [20-22]). In support of this view, GL3 has been shown to be recruited to the promoter region of *CPC *and *ETC1 *[27], and *CPC *has been identified as a direct target gene for WER [26].

**Figure 7:**
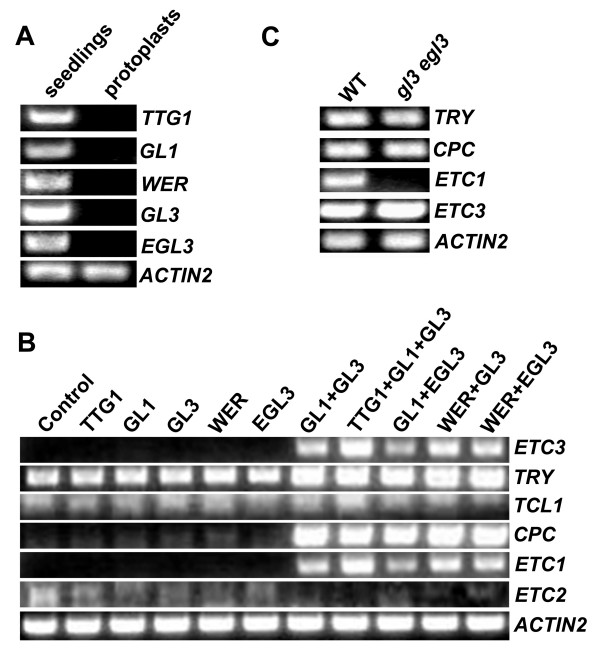
**The regulation of transcription of single-repeat R3 MYB genes by TTG1-GL3/EGL3-GL1 activator complex**. (**A**) Expression of *TTG1*, *GL1*, *WER*, *GL3 *and *EGL3 *in seedlings and protoplasts. RNA was isolated from 10-day-old seedlings or from protoplasts isolated from the rosette leaves of 3-4-week-old plants. RT-PCR was use to examine the expression of *TTG1*, *GL1*, *WER*, *GL3 *and *EGL3*. (**B**) Regulation of single-repeat R3 MYB genes by GL1-GL3/EGL3 and WER-GL3/EGL3. Effector gene(s) were transfected or cotransfected into protoplasts. RNA was isolated from transfected protoplasts that had been incubated for 20–22 h in the darkness. RT-PCR was used to examine the expression of single-repeat R3 MYB genes. (**C**) Expression of single-repeat R3 MYB genes in *gl3 egl3 *double mutant. RNA was isolated from 10-day-old seedlings. RT-PCR was used to examine the expression of *TRY*, *CPC*, *ETC1 *and *ETC3*. The expression of *ACTIN2 *was used as control.

To investigate whether activation of single-repeat R3 MYB genes by the TTG1-GL3/EGL3-GL1/WER complex represents a general regulatory mechanism, we used an Arabidopsis mesophyll protoplast transfection system [32,33]. This system has been successfully used to elucidate the regulatory roles of many other transcription factors [34–38). First, we examined the expression of each component of the activator complex in Arabidopsis rosette leaf mesophyll protoplasts. We found that *TTG1*, *WER*, *GL1*, *GL3 *and *EGL3 *transcripts were undetectable by RT-PCR in wild-type protoplasts, whereas all of them were expressed in the whole seedlings (Figure 7A). These results indicated that Arabidopsis rosette leaf mesophyll protoplasts are suitable for the study of transcriptional activity of these activator complexes by transfection assays because the potential interferences by the endogenous *TTG1*, *WER*, *GL1*, *GL3 *and *EGL3 *are minimized. We also found that the basal transcript level of *TRY *was highest among all single-repeat R3 MYB genes in untransfected protoplasts (Figure 7B). In a preliminary test using Arabidopsis protoplasts from wild-type, we found that GL1 and GL3 are required and sufficient to induce the expression of *CPC *(data not shown). Therefore, in order to simplify our cotransfection assays, we decided to use protoplasts prepared from *ttg1 *mutant rosette leaves to test the activation of single-repeat R3 MYB genes by the GL3/EGL3-GL1 or GL3/EGL3-WER activator complex. To further ensure that the elimination of TTG1 would not impair the transcriptional activity of the activator complex, we compared the transcriptional activation of single-repeat R3 MYB genes by GL1+GL3 or TTG1+GL1+GL3. We found that the GL1+GL3 combination was as effective as TTG1+GL1+GL3 to activate the transcription of the single-repeat R3 MYB genes (Figure 7B). By using this system, we found that none of the components in the proposed activator complexes alone could activate the transcription of any single-repeat R3 MYB genes (Figure 7B). However, GL1 or WER together with GL3 or EGL3 were sufficient to activate the transcription of *TRY*, *CPC*, *ETC1 *and *ETC3*, but not *TCL1 *and *ETC2 *(Figure 7B). These results indicated that some, but not all, single-repeat R3 MYB genes are induced by the proposed activator complexes.

To further investigate if those four single-repeat R3 MYB genes activated by the GL1-GL3/EGL3 or WER-GL3/EGL3 complex, including *TRY*, *CPC*, *ETC1 *and *ETC3*, are tightly regulated by the activator complexes, we examined the expression of these genes in the *gl3 egl3 *double mutant background. We reasoned that if these single-repeat R3 MYB genes are solely regulated by these activator complexes, their expression would be dramatically reduced in *gl3 egl3 *double mutant due to the disruption of the activator complexes. Surprisingly, we found that among these four single-repeat R3 MYB genes, *ETC1 *was the only gene whose transcript was dramatically reduced in the *gl3 egl3 *double mutant (Figure 7C). In order to more accurately compare the transcript level of *TRY*, *CPC *and *ETC3 *between wild-type and *gl3 egl3 *mutant, we used quantitative real-time PCR to quantify the transcript level of these genes. The results from quantitative real-time PCR confirmed that there is no difference in the transcript level of *TRY *and *ETC3 *between wild-type and *gl3 egl3 *mutant whereas the transcript level of *CPC *was only weakly reduced (about 20% reduction) in *gl3 egl3 *mutant.

## Discussion

### Overlapping functions of single-repeat R3 MYB transcription factors in regulating trichome and root hair formation

The six single-repeat R3 MYB transcription factors in Arabidopsis are highly similar to each other at the amino acid level (Figure 1B). However, among these six single-repeat R3 MYB genes, only single mutants of *TRY*, *CPC *and *TCL1 *displayed major phenotypes in epidermal cell development [1-4], whereas single mutants of *ETC1*, *ETC2 *and *ETC3 *are largely indistinguishable from wild-type plants [5-8]. Further, the phenotypes of *try*, *cpc *and *tcl1 *single mutants are distinct. *try *mutant displays characteristic trichome clusters in leaves [1,2], *cpc *mutant has increased trichome formation on leaves and had decreased root hair formation [2,3], whereas *tcl1 *mutant produces ectopic trichomes on inflorescence stems and pedicels [4]. These findings suggested that TRY is a major regulator regulating trichome cluster formation, CPC is a major regulator regulating root hair and trichome formation, whereas TCL1 is a major regulator regulating trichome formation on inflorescence stems and pedicels. Available evidence suggested that single-repeat R3 MYB transcription factors can also function redundantly to regulate trichome and root hair formation [2,4-9]. Each of those previous studies of these single-repeat R3 MYBs has been limited to an analysis of only a subset of these six genes, and furthermore, they have limited their attentions to epidermal development in only one or two of these organs. Here we conducted a comprehensive analysis of the roles of single-repeat R3 MYB transcription factors in trichome and root hair formation. Our comprehensive analysis enables us to draw broader conclusions about the role of this gene family than were possible in the earlier studies.

Mutants generated including the *cpc etc1 etc3 tcl1 *quadruple mutant containing loss-of-function mutations in all members of group I single-repeat R3 MYB genes, and the *try cpc etc1 etc3 tcl1 *quintuple mutant. By analyzing these single, double, triple, quadruple and quintuple mutants, we made several new discoveries. First, we established a role for TCL1 in controlling leaf trichome formation and root hair formation (Figure 2, Table 1, Table 2). TCL1 has been shown to be a major regulator of the single-repeat R3 MYB family controlling trichome formation on the inflorescence stems and pedicels [4], but a role of TCL in controlling trichome formation on leaves and root hair formation had not been previously established. Second, we established a role for ETC1 and ETC3 in controlling trichome formation on the inflorescence stems and pedicles (Figure 3). We note that a recent study showed that a different allele of *etc3*, called *cpl3-1*, was shown to produce about 80% more trichomes and 20% fewer root hairs than wild-type [9], which may be due to an allele-specific difference in *etc3 *effect. Third, we found that mutations in *TCL1*, *ETC1 *and *ETC3 *can increase leaf trichome cluster frequency in *cpc *mutant background (Table 1). About 16% of the trichomes formed in clusters in the *cpc tcl1 etc1 etc3 *quadruple mutant (Table 1). Working together with TRY, they also control trichome cluster formation on the inflorescence stems (Figure 5). Finally, we demonstrated that in addition to regulating trichome formation on leaves and inflorescence stems, single-repeat R3 MYB transcription factors also regulate trichome formation on cotyledons and siliques (Figure 4). Because these organs normally do not bear any trichomes, these results suggested that single-repeat R3 MYBs normally suppress trichome formation on these organs in a highly redundant manner.

### Mechanism of the action of single-repeat R3 MYB transcription factors in the regulation of trichome and root hair formation

The six single-repeat R3 MYB transcription factors are approximately 50% identical to each other at the amino acid level (Figure 1B). More importantly, these six single-repeat R3 MYB transcription factors contain the amino acid signature [D/E]L × 2 [R/K] × 3L × 6L × 3R that has been shown to be required for interacting with R/B-like bHLH transcription factors [28] (Figure 1B). Further, the amino acids within the MYB domain that have been shown to be crucial for cell-to-cell movement of CPC protein [29] are also entirely conserved in all six single-repeat R3 MYB transcription factors (Figure 1B). These results implied that all single-repeat R3 MYB transcription factors have the potential to act in a similar manner by moving from cell to cell to compete with GL1 for binding GL3, thereby limiting the activity of the TTG1-GL3/EGL3-GL1/WER activator complex to regulate trichome and/or root hair formation. Five of these six single-repeat R3 MYB transcription factors, including TRY, CPC, ETC1, ETC2 and ETC3, have been previously shown to interact with GL3 in yeast cells [7,9,17,24,28]. Here we showed that all of these six single-repeat R3 MYB transcription factors interact with GL3 in plant cells (Figure 6), supporting that competition mechanism between single-repeat R3 MYB transcription factors and GL1 for binding GL3 [20-23]. These results suggested that single-repeat R3 MYB transcription factors may mediate lateral inhibition in epidermal cell patterning by a general mechanism: competing with GL1 for binding GL3 to reduce the activity of the TTG1-GL3/EGL3-GL1 activator complexes. It remains to be tested if all single-repeat R3 MYB transcription factors can also directly suppress the expression of *GL1 *thus inhibiting the formation of the TTG1-GL3/EGL3-GL1 activate complex, as has been demonstrated for TCL1 [4].

### Regulation of the transcription of single-repeat R3 MYB genes

Previous studies suggested that the expression of single-repeat R3 MYB genes is regulated by the TTG1-GL3/EGL3-GL1 activator complex in shoots, and by the TTG1-GL3/EGL3-WER activator complex in roots. These single-repeat R3 MYB transcription factors move from a trichome precursor cell to its neighboring cell in shoots, or move from an N cell to an H cell in roots, to compete with GL1 or WER for binding GL3, thus limiting the activity of activator complexes [20-23]. Using an Arabidopsis protoplast transfection system, we found that GL1-GL3/EGL3 or WER-GL3/EGL3 complexes are required and sufficient to activate the expression of *TRY*, *CPC*, *ETC1 *and *ETC3 *(Figure 7B). However, we did not detect the activation of *TCL1 *and *ETC2 *by these complexes (Figure 7B). Moreover, among the single-repeat R3 MYB genes that can be activate by the GL1-GL3/EGL3 or WER-GL3/EGL3 complex, only the transcript of *ETC1 *is dramatically reduced in the *gl3 egl3 *double mutant (Figure 7C). These results implied that: (i) only the transcription of *ETC1 *of the small MYB gene family is tightly controlled by the GL1-GL3/EGL3 and WER-GL3/EGL3 activator complexes; (ii) in addition to being controlled by the GL1-GL3/EGL3 and WER-GL3/EGL3 activator complexes, there are additional mechanisms controlling the transcription of *TRY*, *CPC *and *ETC3*; and (iii) the transcription of *TCL1 *and *ETC2 *are controlled by unidentified mechanisms. Therefore, our results suggested that there may be multiple, distinct mechanisms controlling the transcription of single-repeat R3 MYB genes. The differential regulation of single-repeat R3 MYB genes may contribute to the differences in their developmental functions, which might explain why so many genes with very similar function are maintained in the genome.

## Conclusion

We have established that each of the single-repeat R3 MYB transcription factors has a role in regulating trichome and root hair formation, and that these MYBs largely function in a redundant manner. We demonstrate that a normal function of these MYBs is to suppress trichome formation on leaves and inflorescence stems, and to suppress trichome formation on cotyledons, pedicels and siliques, organs that normally do not bear any trichomes. We confirm that GL3 binding may represent a general mechanism of action of single-repeat R3 MYBs in inhibiting the activity of the TTG1-GL3/EGL3-GL1 activator complex. We show that the transcription of small MYB genes is likely regulated by multiple mechanisms. These results reveal genetic basis of organ-specific control of trichome formation and provide new insight into the lateral inhibition mechanism that mediates epidermal patterning.

## Methods

### Plant materials and growth conditions

The single mutants, *try_29760 *[24], *etc1-1 *[6], *tcl1-1 *[4], *etc2-2 *and *etc3-1 *[8] are in the Columbia-0 (Col-0) ecotypic background. The *cpc-1 *mutant is in the Wassilewskija (WS) ecotypic background [3]. The *ttg1 *and *gl3 egl3 *mutants are in the Landsberg erecta (Ler) ecotypic background [17]. Double mutants were generated by crossing single mutants. Triple, quadruple and quintuple mutants were generated by crossing related lower order mutants (e.g. single, double and triple mutants). Mutants were examined in the F2 progeny for putative mutant phenotype, and their mutant statuses were confirmed by genotyping in F2 and subsequent generations. In this study, *try*, *etc1*, *tcl1*, *etc2*, *etc3 *and *cpc *refer to the specific alleles *try_29760 *[24], *etc1-1 *[6], *tcl1-1 *[4], *etc2-2 *[8], *etc3-1 *[8] and *cpc-1 *[3], respectively.

Seedlings used for RT-PCR analysis were obtained by growing surface-sterilized seeds on 0.6% (w/v) phytoagar (plantmedia, Dublin, Ohio) solidified 1/2 Murashige & Skoog (MS) medium with vitamins (plantmedia) and 1% (w/v) sucrose. Seedling used for phenotypic analysis were obtained either by plating seeds on 1/2 MS medium or by directly sowing seeds into soil. Plants were grown at 23°C with 14/10 h photoperiod at approximately 120 μmol m^-2 ^s^-1^.

### Plasmid construction

Constructs used for protoplasts transfection were generated by first amplifying the full-length open-reading frame (ORF) of the corresponding genes by RT-PCR using RNA isolated from 10-d old, light-grown Arabidopsis seedlings, then cloning the PCR fragment in frame with an amino terminal HA or GD tag into the *pUC19 *vector under the control of the double *35S *enhancer promoter of *CaMV *[36,39]. For plant transformation, corresponding constructs in *pUC19 *vector was digested with *EcoR*I, then sub-cloned into binary vector *pPZP211 *or *pPZP221 *[40].

### Plant transformation and selection of transgenic plants

Five-week-old soil grown plants with several mature flowers on the main inflorescence stem were used to transform with related constructs in *Agrobacterium tumefaciens *GV3101 by the floral dip method [41]. Phenotypes of transgenic plants were examined in the T1 generation, and confirmed in T2 up to T4 generations. For all transgenic plants, at least five transgenic lines with similar phenotypes were obtained.

### Protoplasts isolation, transfection and GUS activity assay

Protoplast isolation, transfection and GUS activity assays were performed as described previously [4,36].

### Microscopy

Trichomes and root hairs were analyzed and photographed as described [4]. The pattern of epidermal cell types was determined as described previously [6,7,42].

### RNA isolation, RT-PCR and quantitative real-time PCR

For RT-PCR, total RNA was isolated from seedlings or transfected protoplasts using the RNeasy Plant Mini Kit (QIAGEN, Mississauga, Ontario, Canada). cDNA was synthesized using 1 μg total RNA by Oligo(dT)-primed reverse transcription using OMNISCRIPT RT Kit (QIAGEN). The primers used for cloning or examining the expression of corresponding genes are as follows: *TRY*-specific primers: 5'-ATGGATAACACTGACCGTCG-3' and 5'-CTAGGAAGGATAGATAG-3', *CPC*-specific primers: 5'-ATGTTTCGTTCAGACAAGGC-3' and 5'-TCATTTCCTAAAAAAGTCCT-3', *TCL1*-specific primers: 5'-ATGGATAACACAAACCGTC-3' and 5'-TCATTTGTGGGAGAAATAGTC-3', *ETC1*-specific primers: 5'-ATGAATACGCAGCGTAAGTC-3' and 5'-TCAACGTAATTGAGATCTTCG-3', *ETC2*-specific primers: 5'-ATGGATAATACCAACCGTC-3' and 5'-TTACAATTTTAGATTTTCTTG-3', *ETC3*-sepcific primers: 5'-ATGGATAACCATCGCAGGAC-3' and 5'-TCAATTTTTCATGACCCAAAAC-3', *TTG1*-specific primers: 5'-ATGGATAATTCAGCTCCAG-3' and 5'-TCAAACTCTAAGGAGCTGC-3', *GL1*-specific primers: 5'-ATGAGAATAAGGAGAAG-3' and 5'-CTAAAGGCAGTACTCAACATC-3', *WER*- specific primers: 5'-ATGAGAAAGAAAGTAAGTAG-3' and 5'-TCAAAAACAGTGTCCATC-3', *GL3*-specific primers: 5'-ATGGCTACCGGACAAAACAG-3' and 5'-AAGGAACGGGAAGCAAACCACTGTG-3', *EGL3 *specific primers: 5'-ATGGCAACCGGAGAAAACAGAACG-3' and 5'-TCTCAAGGACTCCTCCAAGAAACG-3', *ACTIN2 *specific primers: 5'-CCAGAAGGATGCATATGTTGGTGA-3'and 5'-GAGGAGCCTCGGTAAGAAGA-3'. The quantitative real-time PCR was performed using the MJ MiniOpticon real-time PCR system (Bio-Rad, http://www.biorad.com) and IQ SYBR Green Supermix (Bio-Rad).

## Authors' contributions

SW and YC isolated the double, triple, quadruple and quintuple mutants. SW and LH performed trichome and root hair analyses. SW performed the protoplast transfection assays. JG participates in making constructs and performing plant two-hybrid protein-protein assays. JS and J–GC conceived and coordinated the study. All authors participated in drafting and editing the manuscript, and read and approved the final manuscript.
